# ﻿*Primulalongipilosa* (Primulaceae), a new species from Yunnan, China

**DOI:** 10.3897/phytokeys.194.81335

**Published:** 2022-04-11

**Authors:** Ze-Huan Wang, Yi Wang, Li Chen, Hua Peng, Zhi-Kun Wu, Guang Guo

**Affiliations:** 1 Department of Traditional Chinese Medicine Resources and Development, College of Pharmacy, Guizhou University of Traditional Chinese Medicine, Guiyang 550025, Guizhou, China Guizhou University of Traditional Chinese Medicine Guiyang China; 2 Key Laboratory of Forest Plant Cultivation, Development and Utilization of Yunnan Province, Yunnan, China Key Laboratory of Forest Plant Cultivation, Development and Utilization of Yunnan Province Kunming China; 3 Yunnan Key Laboratory of Conservation and Breeding of Rare and Endangered Forest Plants, State Forestry Administration, Yunnan Academy of Forestry & Grassland Science Kunming 650201, Yunnan, China tate Forestry Administration, Yunnan Academy of Forestry & Grassland Science Kunming China; 4 CAS Key Laboratory for Plant Diversity and Biogeography of East Asia, Kunming Institute of Botany, Chinese Academy of Sciences, Kunming 650201, Yunnan, China Kunming Institute of Botany, Chinese Academy of Sciences Kunming China; 5 Forestry and Grassland Bureau, Lincang 677000, Yunnan, China Forestry and Grassland Bureau Lincang China

**Keywords:** Morphological characteristics, nrITS, phylogenetic analysis, *Primulamollis*

## Abstract

*Primulalongipilosa* from SW Yunnan, China, is described as a species new to science and illustrated. The systematic placement of this new species is also discussed based on an nrITS molecular tree. It is morphologically most similar to *P.mollis*, but differs from the latter in its racemose inflorescence, green calyx tube, pink to pink rose corolla, stamens at 1/3 length above the base of the corolla tube and applanate globose capsule.

## ﻿Introduction

*Primula* L. is one of the largest genera of Primulaceae, including ca. 500 species mostly indigenous to the north temperate zone. There are ca. 300 species of 24 sections in China, mostly distributed in western Sichuan, eastern Xizang, and northwestern Yunnan (Hu 1990; Hu and Kelso 1996). SectionCortusoides Balf. f. (39: 140, 1913) of the genus *Primula* comprises ca. 20 species, mainly distributed in Eastern Himalaya and Hengduan Mountain in China. The species of this section can be distinguished by a set of morphological characters: Perennial herb, plants always with multicellular hairs; Leaves entire, shallowly undulate or palmately divided, base cordate or rounded, with long stipe; Inflorescences umbellate in 1–10 whorls, rarely racemose; flowers usually heteromorphic; Calyx narrowly campanulate to tubular, shorter than corolla tube, with many longitudinal veins; Corolla pink to violet; Capsule split into several pieces.

As one of the hotspots of biodiversity in China, Yunnan Province has ca. 130 species of *Primula* distributed all over its range (Fang 2003). The number is still increasing with new taxa constantly being reported in this province over the past two decades (Rankin et al. 2002; Gong and Fang 2003; Xue and Zhang 2004; Shui and Chen 2006; Li and Hu 2009; Hu and Hao 2011; Yang et al. 2017; Wu et al. 2019; Ma et al. 2021).

During the National Survey of Traditional Chinese Medicine Resources field survey in Gengma County, Yunnan province, we discovered one flowering population of *Primula* with distinct long white soft multicellular hairs, racemose inflorescences, and pink corolla. After further morphological studies and molecular phylogenetic analysis, we confirmed that it represents a species new to science, which is described and illustrated here.

## ﻿Materials and methods

### ﻿Morphological analysis

The morphological description of the new species was based on examining the type specimens (KUN) collected from the type locality and corresponding photos taken in the field. We referred to the keys to sections and species in Flora Reipublicae Popularis Sinicae (Hu 1990) and Flora of China (Hu and Kelso 1996). The comparison with morphologically similar species (*Primulamollis*) was based on studies of the descriptions and illustrations in the protologue (Hooker 1854), Flora Reipublicae Popularis Sinicae (Hu 1990), and Flora of China (Hu and Kelso 1996).

### ﻿Taxon sampling and outgroup selection

The phylogenetic analysis was mainly based on the recently published framework of *Primula* (Xu et al. 2016); we performed nuclear nrITS sequencing of *P.longipilosa* and remained all the taxa of subgen. Auganthus (Link) Wendelbo (11: 34, 1961) and subgen. Carolinella (Hemsl.) Wendelbo (11: 36, 1961), added a few species available on GenBank to make our analysis focus on the sect. Cortusoides and sect. Malvacea Balf. f. (39: 145, 1913). *Androsacesublanata* Hand.-Mazz. was used as the outgroup to keep consistent with the former framework. All the sequences downloaded from GenBank (www.ncbi.nlm.nih.gov/Genbank) were marked with the accession number in the phylogenetic tree.

### ﻿DNA extraction, sequencing, and phylogenetic analysis

For the molecular phylogenetic analysis, DNA sequences were newly generated with the protocols described by Wang et al. (2013) for the nuclear ribosomal internal transcribed spacer (nrITS). The GenBank accession number of the new sequence is OM436005. The nrITS dataset was analyzed for phylogenetic tree reconstruction with Bayesian Inference and Maximum Parsimony as described by Wang et al. (2013); SYM + I + G substitution model was selected using jModelTest2 2.1.6 (Darriba et al. 2012) for Bayesian inference (BI) analysis.

## ﻿Results

### ﻿Phylogenetic reconstruction

**Figure 1.**
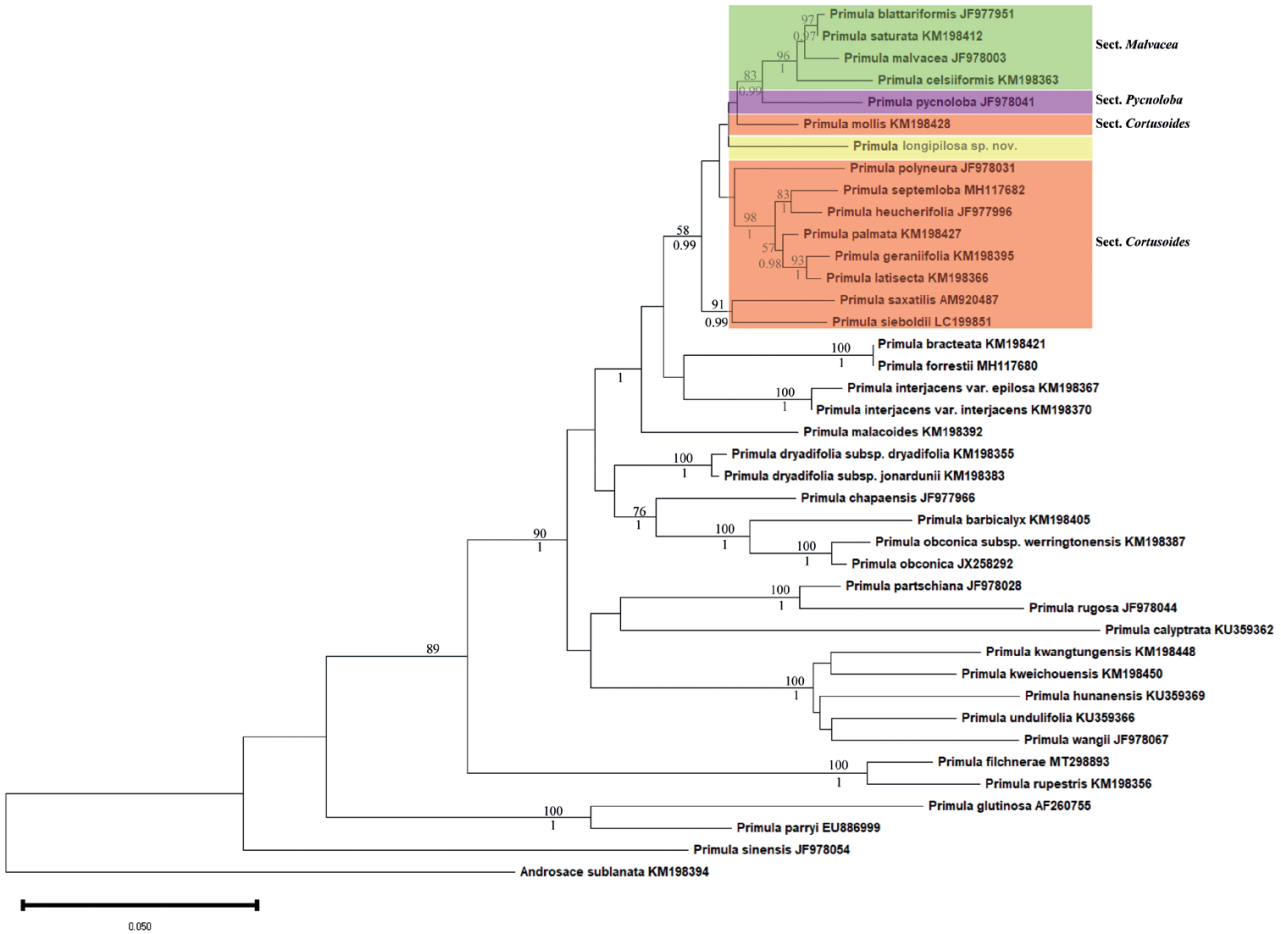
Jackknife 50% majority-rule tree of selected species from *Primula* (Primulaceae) based on the nrITS region, focusing on the subgen. Auganthus and the subgen. Carolinella. The MP jackknife support (JK) values are shown above the branches, and the BI posterior probabilities (PP) are given below the branches.

As the Bayesian and Maximum Parsimony analysis generated similar results, only the Jackknife 50% majority-rule consensus tree is presented here (Fig. 1). The phylogenetic analysis showed that the sequence of the new species nested within the subgen. Auganthus, forming a clade with the sect. Malvacea, sect. Pycnoloba Balf. f. (39: 144, 1913) and *P.mollis* of the sect. Cortusoides with low support value (JK < 50; BI < 0.9). However, if we collapse all the low support nodes, the relationships between the new species with these three sections will remain unresolved.

### ﻿Taxonomic treatment

#### ﻿ Primula longipilosa

Taxon classificationPlantae

Ze H. Wang & H. Peng sp. nov.

F80F3F0A-0ADD-5ADC-9914-8EF578B27919

urn:lsid:ipni.org:names:77296980-1

Figs 2 , 3

##### Type.

China, Yunnan Province, Gengma County, Gengma Town, new Aiguo Village. 23°39.91'N, 99°32.44'E, alt. 1384 m, 31 July 2020, Gengma TCM Resources Survey Exped. 5309260482 (holotype: KUN!, isotypes: KUN!).

**Figure 2.**
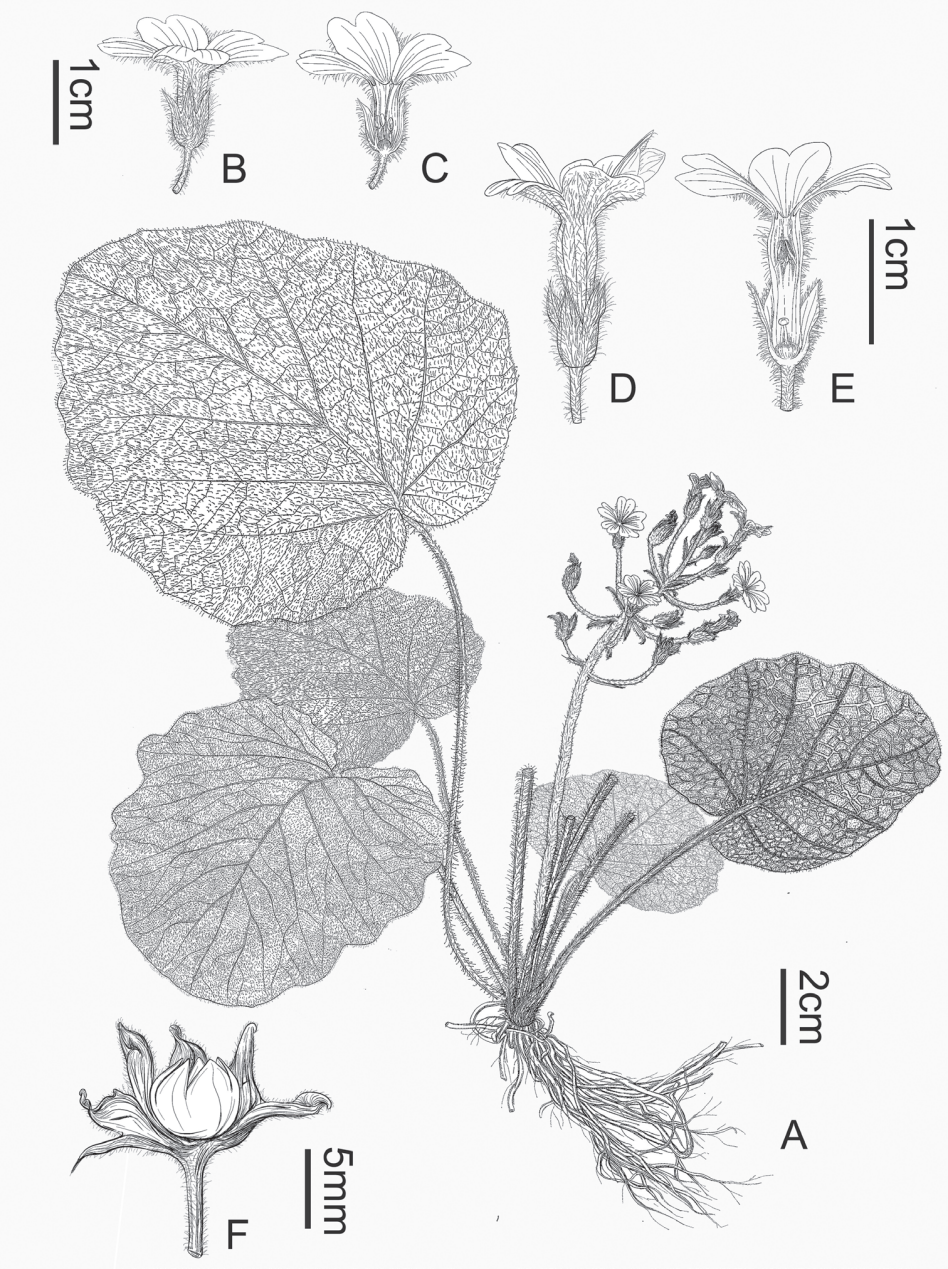
*Primulalongipilosa* sp. nov. **A** habit **B–C** pin flowers **D–E** thrum flowers **F** capsule with dissected calyx. Drew by Dr. Yuan Luo.

##### Diagnosis.

*Primulalongipilosa* is most similar to *P.mollis* in the long soft multicellular hairs all over the plant, the shape of their leaves and corolla. But it differs from the latter mainly in its racemose inflorescence, green calyx tube, pink to pink rose corolla, stamens of the pin flowers at 1/3 length above the base of corolla tube, and applanate globose capsule. The main morphological differences between *P.longipilosa* and *P.mollis* are summarized in Table 1.

**Table 1.** Comparison of the morphological characters between *Primulalongipilosa* and *P.mollis*.

Character	*P.longipilosa*	*P.mollis*
Leave blade	margin undulate	margin sinuate-lobulate and denticulate-crenulate
Inflorescence	racemose, shorter than or almost equal to the leaves	umbellate, umbels 3–10, superimposed, significantly longer than leaves
Pedicel	covered with dense hairs	covered with sparse hairs
Calyx	tube green	tube deep red
Corolla	pink to pink rose	deep red
Stamens of Pin flowers	stamens at 1/3 length above the base of corolla tube	stamens at the middle of the corolla tube
Capsule	applanate globose	Ellipsoid

**Figure 3.**
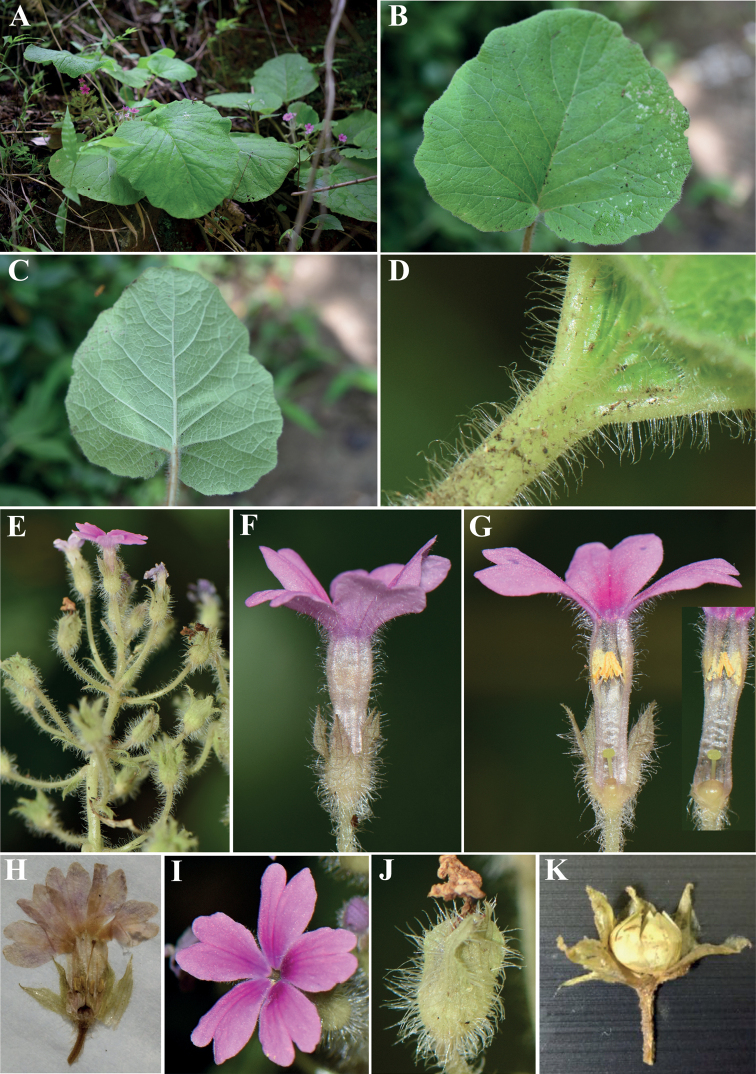
*Primulalongipilosa* sp. nov. **A** habit **B** upper face of leaves **C** lower face of leaves **D** hairs on the petiole **E** racemose inflorescence **F–G** thrum flowers **H** pin flower **I** front side of corolla **J** calyx in late-flowering **K** capsule with dissected calyx. Photographed by Li Chen.

##### Description.

Perennial herbs with several robust fibrous roots. The whole plant is covered with long white soft multicellular hairs. Stem extremely shortened, inconspicuous. Leaves all rising from the root, forming a rosette; petiole 5–20 cm, clothed with long spreading soft multicellular hairs, slightly sheathing at the base; leaf blade cordate to broad cordate, 3.5–19 cm long, 4–16 cm wide, covered with white soft multicellular hairs on both sides; apex obtuse, base cordate or deeply cordate, margin undulate; basal veins usually 3–5, lateral veins 5–6, all raised abaxially and further branched to form reticulate fine veins. Scapes 7–17 cm long, 2–3 rising from the middle of the rosette leaves, shorter than or almost equal to the leaves, densely covered with long soft multicellular hairs, each scape has 7–25 flowers arranged in a racemose inflorescence, or sometimes several nearby flowers grow close to each other to form an umbel in some part of the inflorescence; bracts narrowly lanceolate, 0.5–1 cm long, with long soft multicellular hairs. Pedicel 1.5–2.5 cm, extended to 4 cm in fruit, densely covered with long soft multicellular hairs. Flowers heterostylous. Calyx narrowly campanulate, green, 6–10 mm, covered with long soft multicellular hairs abaxially, parted to the middle; lobes triangulate to ovate-triangulate; veins 3–5. Corolla pink to pink rose, with long soft multicellular hairs outside; tube 1–1.2 cm long; limb ca.1.5–2 cm in diameter; lobes obcordate, ca. 9 mm, with several rays sending out from the mouth, which is prominent especially at the base, bifid at the apex; pin flowers: stamens at ca 3 mm above the base of corolla tube, their style ca 8 mm long; thrum flowers: stamens at 2/3 length of corolla tube, ca. 1 cm above the base of corolla tube, their style ca 2 mm. Ovary applanate globose, stigma a depressed globose disc. Capsule applanate globose, ca. 5 mm in diameter, hidden by the persistent calyx, 5-toothed split.

##### Distribution and habitat.

*Primulalongipilosa* is currently encountered and seen growing on the moist mountain slopes along the valley forest margin near the new Aiguo Village, Gengma County, Yunnan Province, China.

##### Phenology.

Flowering and fruiting from July to August.

##### Etymology.

The specific epithet refers to the impressive long spreading white soft multicellular hairs on the whole plant.

##### Vernacular name.

Simplified Chinese: 长毛报春; Chinese Pinyin: Chángmáo Bàochūn.

##### Threat status.

Currently, the authors have discovered only one population of *Primulalongipilosa* with ca. 30 individuals from the type locality. Some plants grow very close to the path to face a stronger strength from human activities. However, as the authors conducted no detailed field survey for this new species in the adjacent districts, whether some other populations exist remains unknown. Considering its localized distribution in SW Yunnan, its status should nevertheless be of concern and addressed by further investigations.

##### Relationship with related species.

According to the keys of *Primula* in Flora Reipublicae Popularis Sinicae (Hu 1990) and Flora of China (Hu and Kelso 1996), *Primulalongipilosa* should be ascribed to the sect. Cortusoides by a combination of the following morphological characters: long soft multicellular hairs all over the plant; leaves with long stipe, base cordate or deeply cordate, margin undulate; Flowers heterostylous; Calyx narrowly campanulate, veins 3–5; Corolla pink to pink rose.

The molecular phylogenetic study also showed that *P.longipilosa* had a close relationship with the species of the sect. Cortusoides. It was most closely related to *P.mollis* of this section in terms of the long soft multicellular hairs all over the plant, the shape of their leaves and corolla, and also their distribution area. As there are some discrepancies or feature omissions in the description of *P.mollis* in different flora, we referred to its protologue and color illustration for the morphological comparison between them. The detailed morphological comparison between both species is shown in Table 1.

## Supplementary Material

XML Treatment for Primula longipilosa
